# Effects of tDCS on Foot Biomechanics: A Narrative Review and Clinical Applications

**DOI:** 10.3390/bioengineering10091029

**Published:** 2023-08-31

**Authors:** Songlin Xiao, Bin Shen, Chuyi Zhang, Zhen Xu, Jingjing Li, Weijie Fu, Jing Jin

**Affiliations:** 1 School of Exercise and Health, Shanghai University of Sport, Shanghai 200438, China; xiao_songlin@126.com (S.X.);; 2 Key Laboratory of Exercise and Health Sciences of Ministry of Education, Shanghai University of Sport, Shanghai 200438, China; 3 School of Psychology, Shanghai University of Sport, Shanghai 200438, China

**Keywords:** neuro-biomechanical enhancement techniques, foot biomechanical characteristics, foot performance, ergogenic mechanism

## Abstract

In recent years, neuro-biomechanical enhancement techniques, such as transcranial direct current stimulation (tDCS), have been widely used to improve human physical performance, including foot biomechanical characteristics. This review aims to summarize research on the effects of tDCS on foot biomechanics and its clinical applications, and further analyze the underlying ergogenic mechanisms of tDCS. This review was performed for relevant papers until July 2023 in the following databases: Web of Science, PubMed, and EBSCO. The findings demonstrated that tDCS can improve foot biomechanical characteristics in healthy adults, including proprioception, muscle strength, reaction time, and joint range of motion. Additionally, tDCS can be effectively applied in the field of foot sports medicine; in particular, it can be combined with functional training to effectively improve foot biomechanical performance in individuals with chronic ankle instability (CAI). The possible mechanism is that tDCS may excite specific task-related neurons and regulate multiple neurons within the system, ultimately affecting foot biomechanical characteristics. However, the efficacy of tDCS applied to rehabilitate common musculoskeletal injuries (e.g., CAI and plantar fasciitis) still needs to be confirmed using a larger sample size. Future research should use multimodal neuroimaging technology to explore the intrinsic ergogenic mechanism of tDCS.

## 1. Introduction

The foot is a complex structure used in daily activities, such as standing, walking, running, and jumping [1]. The biomechanical function of the foot plays an important role in functional performance, growth, and development, fall risk, and foot disease prevention and treatment [2]. As a structure with direct contact with the ground [3], the foot is one of the most vulnerable parts of the body; the incidence of foot injuries accounts for about 31% [4]. More than one million people are functionally impaired due to foot injuries (flat feet, ankle instability, Achilles tendonitis, plantar fasciitis, etc.) every year, resulting in approximately 1.2 billion in direct health care costs in the United States [5]. Foot injuries are caused by a number of factors, including impairment of biomechanical function [6]. Therefore, strategies to strengthen and restore biomechanical function have become a research hotspot in the fields of sports medicine and rehabilitation.

The latest biomechanical paradigm regards the human foot as a foot core system, which includes active, passive, and neural subsystems [7]. In general, the foot neural subsystem (sensory receptors) collects information (such as force) and transmits it to the cerebral cortex for integration and processing with visual/auditory information to form action instructions, which are fed back to the foot active subsystem (muscles) and ultimately complete a series of motion controls [8,9]. The regulatory function of the central nervous system, which is the core of information integration and processing, is prominent in the foot musculoskeletal system, especially after musculoskeletal injury [10]. Accordingly, musculoskeletal injury is accompanied by adaptive changes in the sensorimotor cortex (SM1) [11]. Additionally, the dyslexic children showed a flat-footed trend and an unstable balance compared with healthy subjects, indicating that cerebellar dysfunction in dyslexia limits the extent of motor control and coordination [12]. Although conventional foot strength training can enhance foot biomechanical function and related physical performance, it cannot effectively regulate the neural circuitry responsible for the neuromechanical regulation of the foot.

In 2016 and 2017, *Nature* reported that transcranial direct current stimulation (tDCS) can enhance the connection between the cerebral cortex and neuromuscular system; this process, which is called “*brain doping*” technology, enables athletes to ultimately improve the jumping force by 70% and their coordination by 80% [13,14]. Since then, tDCS has been applied to enhance human memory, learning, perception, and other abilities and preliminarily introduced into the field of biomechanics to explore potential effects on biomechanical function. Applying tDCS over M1, which controls the foot, is challenging because the feet are the furthest body part from the brain. Limited evidence has shown that tDCS can directly regulate the neural excitability of the cerebral cortex to improve foot biomechanical function and prevent foot injuries [15,16]. As such, the ergogenic effect and clinical application of tDCS in foot biomechanics should be systematically summarized.

This study aimed to summarize research on the effects of tDCS on foot biomechanics and its clinical applications and discuss the potential underlying mechanisms for improving foot biomechanical performance. Results would provide a basis to optimize the implementation of tDCS, enhance the biomechanical characteristics of the foot, and supply theoretical evidence for preventing injuries.

## 2. Methods

### 2.1. Search Strategy

This narrative review was performed for relevant papers from the first data available until July 2023 in the following databases: Web of Science, PubMed, and EBSCO. The search was performed using the terms “foot biomechanics”, “biomechanical characteristics”, “foot biomechanical characteristics”, “foot biomechanical function”, “toe biomechanics”, “ankle biomechanics”, “foot”, “toe”, and “ankle” which were separately combined with “transcranial direct current stimulation” or “tDCS” in all databases. Boolean operators “AND” and “OR” were used to combine keywords according to the recommendations of each database. All results found in the search were imported into the EndNote reference manager (EndNote X9, Stanford, CA, USA) to gather together and automatically find duplicate records.

### 2.2. Eligibility Criteria and Article Selection

The inclusion criteria were as follows. (1) The participants were healthy adults or patients with lower limb musculoskeletal injury. (2) Intervention was tDCS or combined interventions, regardless of stimulation types, stimulus intensity, duration, and electrode location. (3) The primary outcomes were biomechanical characteristics, including strength, perception, flexibility, or other related variables of the foot and ankle. Animal studies and non-English studies were excluded. Reviews, case reports, letters, opinions, and conference abstracts were also excluded.

A total of 203 related articles were found in the databases (58 in PubMed, 53 in EBSCO, and 92 in Web of Science). Only sixteen articles were included for review after removing duplicate articles and excluding irrelevant studies by reading the titles, abstracts, and full texts. As shown in Table 1, a total of 368 participants, consisting of 247 males and 121 females, were recruited with an age of between 18 and 68.8 years. Thirteen studies recruited healthy participants and three studies included patients with lower limb musculoskeletal injury (chronic ankle instability [CAI] and plantar fasciitis).

## 3. Discussion

### 3.1. Effects of Conventional Functional Training on Foot Biomechanical Characteristics

Strengthening intrinsic foot muscles is the main method used to enhance the biomechanical function of the foot. Interventions for enhancing foot muscle strength include flexion of the interphalangeal joint and the metatarsophalangeal joint [31], fast plantarflexion strength training [32], towel curl [33], short foot exercise [33,34], strength training combined with rehabilitation training [35], and neuromuscular electrical stimulation [36]. After at least four weeks of training, the performance of toe flexion and extension improved [37], the maximum autonomous contraction moment of the foot plantar flexor increased [32], the foot flexor strength increased, and the length of the left and right foot arches (static standing position) decreased; moreover, the distance of the left and right single-leg jumps and vertical jump height increased [31]. Participants performed better in functional balance and stretching tasks after short foot exercises [34]. Participants had a larger reduction in the movement of the pressure center in non-dominant limbs during dynamic balance tests than during towel curling [33].

Minimal shoe training is designed to simulate barefoot running training, which can improve intrinsic foot muscles, such as the strength and volume of the flexor digitorum brevis [38]. Minimal shoe training increases the intrinsic foot muscle strength by reducing the mechanical support for the arch [38,39]. Miller et al. [38] randomly divided 33 participants into a minimal shoe group and a traditional running shoe group and subjected them to 12 weeks of running training. The volume of flexor digitorum brevis muscle increased by 11% and 21% in both groups, respectively. The minimal shoe group had significant increases in the area and volume of the abductor digiti minimi (18% and 22%, respectively) as well as longitudinal arch stiffness (60%). After six months of training, the leg and foot muscle area of the runners significantly increased [40].

Specific activities (e.g., aerobic dance, ballet, and gymnastics) can enhance foot muscle strength [41]. After 12 weeks of aerobic dance training, the balance control and plantar foot pressure of the participants significantly improved. In particular, the leg muscle strength and balance control were significantly higher on a small trampoline than on a hard wood surface. Similar to aerobic dance, ballet, and gymnastics training could improve foot biomechanical characteristics, such as foot muscle strength [42,43]. Hence, the above-mentioned interventions can enhance the biomechanical function of the foot by strengthening its muscle strength.

Researchers have increasingly focused on foot core exercise (FCE), which is a set of strength training exercises designed to improve the biomechanical contribution of the foot by strengthening the intrinsic/extrinsic foot muscles. FCE and minimal shoe training can effectively increase the volume and strength of the intrinsic foot muscles [44]. Conventional functional training or exercise ignores the contribution of foot sensory function (the neural subsystem) and fails to affect the neural circuitry responsible for the biomechanical regulation of the foot and ankle.

### 3.2. Effects of tDCS on Foot Biomechanical Characteristics

Non-invasive brain stimulation regulates brain activity by placing electrodes (or magnetic coils) on the outside of the brain without breaking the skin or placing them inside the brain. tDCS is one of the most widely used techniques. It can modulate cortical excitability by delivering low-amplitude current flow between two or more electrodes placed on the scalp, thereby altering the resting membrane potential to induce depolarization or hyperpolarization and modifying the excitability and activity of spontaneous neurons [45]. The effects of tDCS-induced changes in membrane polarity depend on current intensity, which is commonly set to 1−2 mA [46]. Anodal tDCS can induce neuronal excitation, whereas cathodal tDCS inhibits it. In this context, the anode electrode serves as the entry point for positive current into the body, establishing a positive voltage relative to the cathode electrode. The cathode electrode acts as the exit point for positive current from the body. In 2000, Nitsche et al. [45] found that the excitability of the motor cortex can be regulated by applying a weak direct current through the scalp (increased by 40%); the excitability lasted over several minutes after the current stimulation. This study is the first to systematically confirm that tDCS could be used as an effective non-invasive tool for regulating brain excitability. A large number of studies have confirmed the safety and effectiveness of tDCS and applied it to rehabilitation, sports training, and other fields. The safety of tDCS has been verified through animal experiments and clinical studies. Many researchers explored the potential of tDCS in improving physical performance and reported that it can delay fatigue, increase muscle strength, promote motor skill learning, and induce long-term improvement effects [47,48]. tDCS can also improve foot biomechanical characteristics, such as the vibrotactile threshold of the foot sole [17], tactile threshold [18], toe flexor strength, and toe pinch force [24].

An earlier study reported that a single session of 2 mA tDCS applied over the primary motor cortex (M1) for 10 minutes significantly increased the toe pinch force [24]. Cathodal tDCS with the same stimulation parameters improved the range of motion of dorsiflexion [26]. Single-session high-definition tDCS (HD-tDCS) also improved the flexor strength of the first toe, passive ankle kinesthesia, and static standing balance performance [19]. Furthermore, two sessions of bilateral tDCS were an effective method for improving jump height by modulating ankle and knee net energy [29]. In addition, 4 weeks of HD-tDCS and FCE induced distinct benefits on foot sensorimotor function and standing postural control performance in healthy young adults [22]. These findings suggest that tDCS, to some extent, can serve as an effective intervention for enhancing foot movement performance. However, previous studies only examined the effects of intervention targeting peripheral or central elements on the sensorimotor function of the foot. Interventions simultaneously targeting peripheral and central elements could induce greater benefits than traditional “single-target” interventions [49]. Therefore, a novel intervention targeting the peripheral and central elements of sensorimotor regulation was designed by combining FCE and tDCS (i.e., combined intervention). We found that 4 weeks of the combined intervention effectively enhanced the biomechanical function of the foot (i.e., toe flexor strength and passive ankle kinesthesia) [21].

Compared with conventional functional training, tDCS demonstrates advantages in enhancing foot motor performance and has the potential to promote sensory function. For example, Zhou et al. [17] found that 20 min of single-session anodal tDCS significantly reduced the vibrotactile threshold of the foot sole in older adults under weight-bearing conditions. Cathodal tDCS applied over the left M1 area decreased the tactile threshold of the left center of the distal pulp of the hallux [18]. Additionally, a single session of tDCS in isolation appears to produce immediate effects on healthy participants’ sensorimotor function [20]. To our knowledge, foot sensorimotor function, especially the ability to precisely regulate muscle force, plays an important role in maintaining balance when standing, walking, and running [50]. Appropriate force control of the ankle requires the proper functioning of baroreceptors and the regulation of cortical networks of the brain [51]. A single session of HD-tDCS applied over SM1 enhanced force control by modulating the beta-band activity of the sensorimotor cortex [23]. Moreover, tDCS can modulate cortical excitability to influence the neuromuscular reflex, eventually altering the reaction time for dorsiflexion [25]. Shah et al. [27] and Sriraman et al. [28] investigated the effects of tDCS on more complex tasks involving plantarflexion−dorsiflexion control. The former found that cathodal or anodal tDCS targeted the cerebellum; anodal tDCS targeting the M1 improved the accuracy of plantarflexion−dorsiflexion visuomotor tasks. The latter also improved the visuomotor tasks induced by anodal tDCS targeting M1. More importantly, in terms of improving sensory function, HD-tDCS demonstrated more significant improvements in foot sensory function than FCE, particularly in significantly reducing the kinesthetic threshold of inversion and eversion [22].

### 3.3. Clinical Applications of tDCS in Foot Biomechanics

Traditional rehabilitation training can effectively restore functional impairments in patients with musculoskeletal injuries. However, recent neurophysiological evidence suggested that changes in the plasticity of the central nervous system were due to sensory dysfunction, the bilateral nature of joint dysfunction, and prolonged altered motor control caused by musculoskeletal injury [11]. Many studies have begun to focus on adaptive changes in the cerebral cortex after ankle and knee ligament injuries; such changes include a short-term increase in afferent nerve activity caused by pain and swelling after ligament injury and long-term impairment of proprioceptive sensations due to the loss of peripheral afferent information [52]. These dysfunctions result in the reorganization of cortical functional areas in the brain, thereby reducing the ability of the central nervous system to respond promptly to unexpected events. tDCS can help change the excitability pattern of dysfunction in the brain, revert the control ability of the central nervous system by inducing neuronal plasticity, and effectively improve the biomechanical function of the foot [53], as well as further bring a benefit to postural control.

A case report found that 5 consecutive days of tDCS (2 mA, 20 min) decreased the pain intensity and pain-related anxiety in a diabetic patient with plantar fasciitis [54]. After recruiting a sufficient number of participants, a study found that the stimulation protocol improved the foot function index [30]. Relevant animal experiments also partially confirmed the findings. Researchers established a chronic pain model by injecting a drug into the ankle joint of the rat and found that tDCS effectively restored mechanical allodynia and thermal hyperalgesia and its effects lasted two weeks [55].

Two studies focused on the effect of tDCS on the rehabilitation of patients with chronic ankle instability (CAI) by using a combined intervention model. Specifically, 4 weeks of anodal tDCS (2 mA, 20 min) combined with eccentric strength training improved the dynamic balance stability, ankle functional performance, and perceived disablement in individuals with CAI [15]. Another study used HD-tDCS (anode position: Cz, 2 mA, 20 min) combined with short-foot exercise for a 4-week intervention. The dynamic balance performance and proprioception were improved after intervention in individuals with CAI [16]. These randomized controlled studies provided evidence that tDCS can enhance foot biomechanical characteristics in individuals with CAI. However, further studies are needed to explore the effectiveness of tDCS and investigate its neuroregulatory mechanism in improving the biomechanical characteristics of the foot in healthy and injured people [16].

### 3.4. Possible Mechanisms of Foot Biomechanical Responses Induced by tDCS

The underlying mechanism of functional performance improvement induced by tDCS remains unclear but is mainly concentrated in the following aspects: when tDCS starts, neurons are exposed to an external electric field for an instant; the external electric field causes ion displacement inside the neuron, changing the internal charge distribution and cell membrane potential and, eventually, inducing membrane depolarization or hyperpolarization [56,57]. In the initial stage of tDCS (about 3 min), the direct effect of the electric field formed on blood vessels and neural networks is dominated by hemodynamic responses [58], followed by electric field-induced responses in neurovascular coupling [59]. In the middle stage of tDCS, the aftereffects of neurons lead to changes in cortical spinal excitability and hemodynamic responses [57]. Finally, the aftereffects of the electric field may continue to exist for several minutes or even hours after stimulation. The plasticity of synaptic connections may be reformed and enhanced [60], and repeated stimulation may produce cumulative effects [61].

tDCS can enhance cortical excitability, which potentially strengthens synaptic connections between activated neural structures and increases the synchronization of motor unit firing, thereby potentially improving motor performance [62,63]. However, applying tDCS over M1 is challenging because regions that control the foot biomechanical function are located in the deeper longitudinal fissures of the central sulci gyri [64]. Nonetheless, some studies indicated that tDCS can effectively modulate the cortical excitability of the lower limb areas, as proved by an increase in the motor-evoked potentials of the extrinsic foot muscles, such as the tibialis anterior muscle [65]. tDCS also modulated corticospinal excitability in the lower limb region of the motor cortex and enhanced foot biomechanical performance [24,27]. Our recent findings suggested that participants exhibited a greater average percent decrease in beta task-related power spectral density (i.e., greater cortical activation) following a greater percent reduction in the root mean square of force control task (i.e., greater improvement in ankle force control task) after application of HD-tDCS; hence, HD-tDCS could modulate beta-band brain activity to improve ankle force control task [23]. Regarding improvements in foot neuro-biomechanical function, existing research suggests that tDCS primarily enhances excitability in the primary sensory cortex (S1). As S1 is located adjacent to the M1, tDCS applied over M1 can also partially enhance sensory function [17]. Previous studies confirmed that tDCS can augment activation in the left posterior central parafollicular lobule, including S1, thereby modulating and enhancing cortical responses to foot stimulation in healthy adults [66].

Cathodal tDCS can improve foot tactile perception and ankle range of motion [18,26]. Although cathodal tDCS can reduce cortical excitability, its application to the motor cortex on one side can increase the excitability of the opposite side cortex [67] and improve the biomechanical performance of the ipsilateral foot [18]. The decrease in cortical excitability caused by cathodal tDCS may reduce pain sensation and enhance the ankle joint range of motion [26]. Based on current findings, anodal and cathodal tDCS can regulate cortical excitability and ultimately affect foot neuro-biomechanical characteristics. Some studies found that physical performance improved without significant changes in cortical spinal excitability [68,69]. Therefore, the mechanism underlying the ergogenic effect of tDCS should be interpreted with caution.

In summary, in the neural-muscular control pathway of the foot, the central cortical control region, along with multiple secondary neural centers, possibly mediate and integrate the effects of tDCS. These interconnected neural control centers operate at various levels. Consequently, tDCS may excite specific task-related neurons and regulate multiple neurons within the system (including task-related brain networks and secondary neural centers), thereby affecting foot biomechanical characteristics [70].

## 4. Summary

The concept of the foot core system provided a new paradigm for understanding the complex structure and biomechanical characteristics of the foot and its subsystems to provide stability and flexibility and cope with changing foot demands. tDCS has a positive effect on improving foot biomechanical characteristics, including proprioception, muscle strength, reaction time, and joint range of motion. tDCS can be effectively applied in the field of foot and ankle sports medicine and combined with functional training to effectively improve foot biomechanical performance in individuals with CAI. Hence, tDCS can effectively improve foot biomechanical characteristics and serve as an effective rehabilitation method for patients with foot and ankle injuries. The ideal intervention protocol in the future should enhance the ability of the central nervous system to regulate foot biomechanical function while strengthening intrinsic and extrinsic muscle strength.

The efficacy of tDCS applied in the rehabilitation of common musculoskeletal injuries (e.g., CAI and plantar fasciitis) still needs to be confirmed with a larger sample size. Previous studies provided evidence about the possible mechanisms of tDCS; that is, tDCS may excite specific task-related neurons and regulate multiple neurons within the system, ultimately affecting foot biomechanical characteristics. However, the underlying mechanisms of tDCS in improving foot biomechanical performance remain unclear, especially for different specific tasks. Future research should use multimodal neuroimaging technology to explore the intrinsic ergogenic mechanism of tDCS.

## Figures and Tables

**Table 1:** Main characteristics of the included studies.

Study	Participants, Gender, Age (Years)	Anodal/Cathodal Location	Electrode Size (cm^2^)	Current (mA)	Session, Duration (min)	Main Outcomes of Biomechanical Characteristics
Zhou, et al., 2018 [17]	Healthy, 20 M, 61 ± 4	A: Left C3 R: Right supraorbital region	35	2.0	One session, 20	↓Standing vibratory threshold of foot sole
Yamamoto, et al., 2020 [18]	Healthy, 10 M, 22–34	C: Left C3 R: Right supraorbital region	35	1.5	One session, 10	↓Tactile threshold of distal pulp of the hallux
Xiao, et al., 2020 [19]	Healthy, 14 M, 22.8 ± 1.2	A: Cz R: C3, C4, Fz, Pz	1	2.0	One session, 20	→Foot flexor strength →Ankle kinesthesia threshold
Lerma-Lara, et al., 2021 [20]	Healthy, 53 M/54 F, tDCS (22 ± 2), control (23 ± 3)	A: M1 R: Supra-orbital region	35	2.0	One session, 20	↑Pressure pain threshold ↑Electromyographic activity in the lower limb
Xiao, et al., 2022a [21]	Healthy, 30 M, tDCS (20.5 ± 1.8), control (21.3 ± 1.8)	A: Cz R: C3, C4, Fz, Pz	3.14	2.0	Twelve sessions, 20	↑Toe flexor strength ↓Ankle eversion kinesthesia threshold
Xiao, et al., 2022b [22]	Healthy, 36 M, tDCS (21.9 ± 2.1), control (23.5 ± 1.5)	A: Cz R: C3, C4, Fz, Pz	3.14	2.0	Twelve sessions, 20	↑Metatarsophalangeal joint flexor strength ↓Ankle inversion and eversion kinesthesia thresholds
Xiao, et al., 2023 [23]	Healthy, 8 M/8 F, 25.4 ± 1.8	A: Cz R: C3, C4, Fz, Pz	3.14	2.0	One session, 20	↑Ankle plantarflexion force control
Tanaka, et al., 2009 [24]	Healthy, 8 M/2 F, 20–35	A and C: M1 (“hotspot” of the TA muscle) R: Right forehead	35	2.0	One session, 10	↑Toe pinch force
Devanathan, et al., 2016 [25]	Healthy, 6 M/8 F 20–32	A: M1 (“hotspot” of the TA muscle) R: Right supraorbital region	12.5	1.0	One session, 15	↓Ankle dorsiflexion choice reaction time
Mizuno, et al., 2017 [26]	Healthy, 10 M, 25 ± 3	A and C: Cz R: Center of the forehead	35	2.0	One session, 10	↑Ankle range of motion
Shah, et al., 2013 [27]	Healthy, 5 M/3 F, 18–26	A and C: M1 (‘hotspot’ of the TA muscle), left cerebellum R: Right supraorbital region, left buccinator muscle	8	1.0	One session, 15	↑Accuracy index of ankle tracking
Sriraman, et al., 2014 [28]	Healthy, 4 M/8 F, 20–32	A: M1 (“hotspot” of the TA muscle) R: Right supraorbital region	8	1.0	One session, 15	↑Accuracy index of ankle tracking
Zhu, et al., 2023 [29]	Healthy, 15 M, 19.47 ± 1.6	A: C3, C4 R: Ipsilateral shoulders	35	2.0	Two sessions, 20	↑Jump height ↓Maximum ankle torque ↓Ankle positive energy and net energy decreased in the sham condition
Bruce, et al., 2020 [15]	CAI, 9 M/17 F, 18–40	A: M1 R: Right forehead	15	1.5	Ten sessions, 18	↑Dynamic balance and muscle activation ↑Functional performance on a side-hop test ↓Global ratings of perceived disablement
Ma, et al., 2022 [16]	CAI, 15 M/15 F, 18–30	A: Cz R: C3, C4, Fz, Pz	3.14	2.0	Twelve sessions, 20	↓Joint position senses absolute error at 15° inversion ↑Y-balance reach distance
Concerto, et al., 2016 [30]	Plantar fasciitis, 4 M/6 F, 68.8 ± 3.3	A: C1, C2 R: Supraorbital area contralateral to the stimulated area	35	2.0	Five consecutive sessions, 20	↓Pain intensity ↓Foot function index

Notes: A: anodal; C: cathodal; R: reference electrode; M/F: male/female; M1: primary motor cortex; TA: tibialis anterior; tDCS: transcranial direct current stimulation; CAI: chronic ankle instability; ↓: denotes a decrease; **↑**: denotes an increase; **→**: denotes no significant difference. C1, C2, C3, C4, Fz, Pz, Cz: the electrodes placement of the 10/20 EEG system.

## Data Availability

Not applicable.
